# FAIR and Effective Communication of Data on Chemical Contaminant Biotransformation in the Environment

**DOI:** 10.1021/acs.estlett.5c00753

**Published:** 2025-10-22

**Authors:** Stephanie L. Rich, Jasmin Hafner, Moritz Salz, Mojtaba Qanbarzadeh, Fanshu Geng, Liqing Yan, Jinxia Liu, Damian E. Helbling, Christopher P. Higgins, Kathrin Fenner

**Affiliations:** † Department of Chemistry, University of Zürich, 8057 Zürich, Switzerland; ‡ Eawag, Swiss Federal Institute of Aquatic Science and Technology, 8600 Dübendorf, Switzerland; § Department of Civil and Environmental Engineering, 3557 Colorado School of Mines, Golden, Colorado 80401, United States; ∥ School of Civil and Environmental Engineering, 5922 Cornell University, Ithaca, New York 14850, United States; ⊥ Department of Civil Engineering, 5620 McGill University, Montreal, QC H3A 0C3, Canada; # Department of Civil and Environmental Engineering, Hong Kong Polytechnic University, Kowloon, Hong Kong SAR

**Keywords:** Anthropogenic chemicals, biotransformation, FAIR, PFASs

## Abstract

Anthropogenic chemicals and their transformation products are increasingly found in the environment, with persistence being a major driver of chemical risk. Methods for predicting biotransformation products and dissipation kinetics are needed to help regulators identify potentially persistent chemicals and prevent their release to the market and eventually to the environment. Leveraging machine learning and artificial intelligence is a promising avenue to tackle this problem. However, predictive models are only as good as the data used to train them, calling for large, high-quality data sets of biotransformation pathways and kinetics, which are currently lacking. The objectives of this Global Perspective are to (i) emphasize the importance of effectively communicating biotransformation data on chemical contaminants in the environment, (ii) describe specific components of reporting biotransformation pathways in a findable, accessible, interoperable, and reusable (FAIR) format, and (iii) provide a standardized tool for researchers to use for reporting their biotransformation data, with the intent to boost the quality and quantity of available biotransformation data. We demonstrate the application of our reporting tool for the case of perfluoroalkyl and polyfluoroalkyl substances (PFASs) as a means to develop a PFAS biotransformation database, thereby illustrating how the research community could profit from standard biotransformation data reporting.

## Graphical abstract

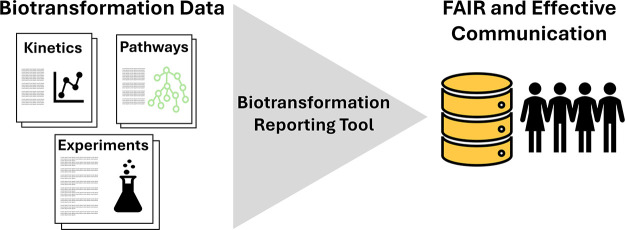

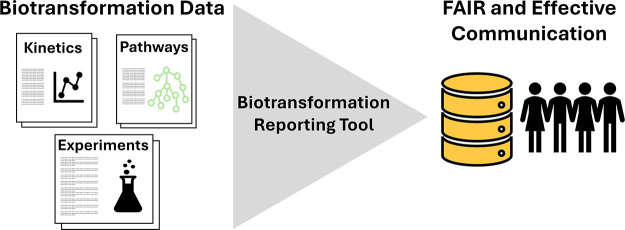

## Introduction

Chemical contaminants released into the environment can be transformed via biological processes, generating biotransformation products that may be more harmful than their precursors.^1^ Despite decades of research on the biotransformations of chemical contaminants in the environment, accurately predicting their biotransformation products and dissipation kinetics remains a challenge. Yet such predictive models are of urgent need for industry and regulators alike, to eventually prevent the late discovery of highly problematic cases such as the accumulation of perfluoroalkyl acids (PFAAs) from the degradation of per- and polyfluoroalkyl substances (PFASs) in the environment.^2^

Models that reliably predict biotransformation product structures and contaminant half-lives in the environment require large, high-quality, machine-readable training data sets with detailed experimental parameters.^3^ However, available data sets are often limited in size and coverage of the chemical space (e.g., specific to pesticides^4^ or hydrocarbons^5^), they lack information on physical, chemical, and biological conditions in the test system,^5^ or they only report dissipation kinetics without pathway information.^6^ Yet, such data are needed to model and predict biotransformation processes and to understand the impact of environmental parameters on these processes.

Data on biotransformation processes can be found in the scientific literature thanks to the increased availability of high-resolution mass spectrometry and regulatory pressure to include hazardous transformation products in chemical risk assessment. Increasing interest in the field of contaminant biotransformation, especially for polyfluoroalkyl substances (*i*.*e*., PFAA precursors), is rapidly increasing the number of literature-reported biotransformation pathways and biotransformation kinetics data sets, which could be used for meta-analysis and model development.^7,8^ Unfortunately, study results are rarely reported in a machine-readable format, posing a major obstacle to data utilization.

The environmental science community can best address this challenge by reporting data in a Findable, Accessible, Interoperable, and Reusable (FAIR) format.^9^ FAIR data principles have been widely accepted across various disciplines and institutions, including the European Commission and the U.S. National Institutes of Health, and will continue to be encouraged as the amount of openly available data increases.^10–13^ This perspective highlights the urgent need for FAIR data sets describing biotransformation pathways and kinetics of chemical contaminants in the environment and provides a tool for reporting biotransformation data in a standardized, machine-readable format. We discuss the needs of data scientists and biotransformation modelers as well as obstacles for experimentalists in data reporting and present a standardized approach for data sharing, including a publicly available tool in the form of a template for this purpose. Our template provides guidance on sharing chemical structures, representing graphical reaction networks, and collecting experimental metadata for best practices in retaining the utility of reported biotransformation data. To highlight the use of this tool for efficient and systematic data reporting, we used it to collect data for polyfluoroalkyl substance biotransformations, and we provide these data in the online, publicly available platform enviPath.^14,15^ We use the example of the enviPath-PFAS data package to demonstrate the utility of aggregating data across studies and to answer relevant questions on the environmental fate of PFASs and highlight prominent data gaps. Moreover, we recognize the importance of community-driven efforts by inviting the research community to contribute to the development of our biotransformation reporting tool and share data publicly in our recommended format. Our recommendations meet cheminformatic standards and provide a basis for future analyses of biotransformation pathways and kinetics in the environment. Finally, the template can be used to publish biotransformation data in enviPath, which implements and promotes FAIR principles, enabling efficient data usage and sharing within the field of biotransformation research.

## Reporting Biotransformation Pathways

Conventional reporting of chemical contaminant biotransformation typically includes pathway figures consisting of 2D images of reactant and product compounds connected by arrows representing singular reaction steps. This visual representation is important for understanding and communicating structural changes to the molecule upon each reaction step, but the reported images are generally not easily translated into a machine-readable format. Given the large number of pathway figures found in the literature, it is extremely challenging to gain a broad overview of the environmental biotransformation processes. The first effort to systematically organize this information was in 1995 through the University of Minnesota Biocatalysis/Biodegradation Database, which has since evolved into the enviPath platform.^16,17^ The enviPath team has continued to develop databases by transcribing pathway and kinetic information from the literature and regulatory documents into an electronic format in a laborious and time-consuming process. This bottleneck in data acquisition prevents researchers from accessing the most up-to-date pathway information. Even though future advances in artificial intelligence (AI) may automate data extraction from study reports, such tools will need high-quality, ground-truth data sets for model training and validation. Therefore, reporting biotransformation data in a standardized format is and will remain relevant for the foreseeable future.

### Recommendation for Standardized Reporting of Biotransformation Data

Standard data reporting practices for future scientific publications are needed to enable more efficient data extraction and aggregation.^18^ We recognize that biotransformation pathways can be complex, and the best way to communicate this information to readers may be nuanced and up to the discretion of the authors. However, we encourage authors to also provide pathway information in a machine-readable format to be submitted alongside the manuscript as Supporting Information. Recommendations and templates for standardized, machine-readable reporting of chemical reaction data have already been provided elsewhere.^18,19^ Given the specific complexity of biotransformation data, we have additionally developed a Biotransformation Reporting Tool (BART) as a Microsoft Excel template to assist authors with reporting their biotransformation data in a FAIR and effective way. BART is freely available on GitHub (https://github.com/FennerLabs/BART). BART has tabs for four different types of information: (i) In the *Compounds* tab, compound structures should be reported as simplified molecular input line entry specifications (SMILES); (ii) The *Connectivity* tab contains the pathway structure as a list of biotransformations, by indicating reactants and products in a tabular format; (iii) Information on the experimental setup and on environmental conditions goes to the *Scenario* tabs; and (iv) Biotransformation kinetics and identification confidence is provided in the *Kinetics_Confidence* tab. An example of how a biotransformation pathway visualization is translated into a tabular format within the *Connectivity* tab is provided in Figure 1, showing the biotransformation of 5:3 fluorotelomer carboxylic acid (5:3 FTCA) into perfluorohexanoic acid (PFHxA) in a bioreactor seeded with aerobic activated sludge.^20^ Figure 1 shows biotransformations with only one reactant and one product each, while the *Connectivity* tab allows for reporting of multiple products. The template also allows flagging biotransformations as *multistep reactions*, where multiple enzymatic steps are hypothesized but not fully elucidated. With mass spectrometry-based transformation product analysis, there is the specific challenge of reporting stereoisomeric transformation products whose structure cannot be fully resolved (e.g., multiple possible positions for hydroxylations on an aromatic ring). In this case, the authors should attempt to draw each alternative structure (*i*.*e*., isomer) within the pathway. For BART reporting, we recommend identifying the most plausible structure based on known biotransformations and indicate it as the primary structure. Up to three alternative structures can be added per compound, and they are designated as alternative structures in the *Compounds* tab. Additionally, compounds identified using mass spectrometry should be annotated with Schymanski Confidence Levels or, when appropriate, PFAS Confidence in Identification (PCI) Levels.^21,22^ Both of these attributes can be reported in the BART template under the *Kinetics_Confidence* tab.^21^ Under the *Scenario* tab, BART further provides lists of key experimental parameters that are frequently collected during experimentation and reported with the pathway information (see Table 1 and the text in the following for details).

**Figure 1:**
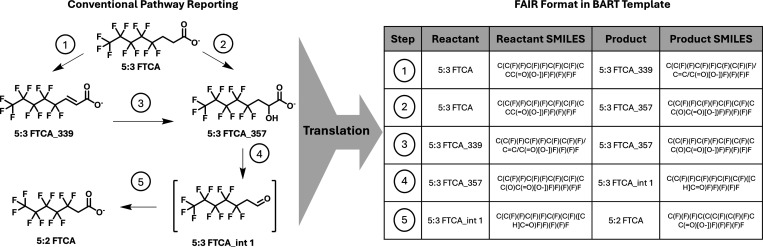
Example of translation process from a documented pathway of 5:3 FTCA biotransformation reported in Geng and Helbling 2024 into the BART template.^20^ Each of the five labeled transformation steps corresponds to a row in the table on the right-hand side of the figure.

We recommend downloading the BART template from our repository and using it to collect and report relevant biotransformation data for experimentally determined pathways if possible. For reactions other than biotransformations or where metadata reporting is not possible or warranted, the more general reaction reporting template proposed by Schymanski et al.^19^ could be used instead. In any case, the filled-out template can be included as Supporting Information for publication with manuscript submissions to scientific journals or uploaded to Zenodo.

**Table 1:** List of Key Parameters to Report for Testing the Biotransformation of Chemicals in Sludge, Soil, and Water–Sediment Systems and General Parameters That Are Relevant for All Test Types^a^

	General	Sludge	Soil	Sediment
Inoculum provenance	Sample location	Biological treatment technology	Soil origin	Sampling depth
		Inoculum source		Sediment origin
		Purpose of WWTP		
		Solids retention time		
				
Sample description	Ammonia uptake rate	**Dissolved organic carbon**	**Bulk density**	**Microbial biomass in sediment**
	**Organic content**	**Dissolved oxygen concentration**	**Cation exchange capacity (CEC)**	**Cation exchange capacity (CEC)**
	Redox condition	**Nitrogen Content**	**Microbial biomass**	**Microbial biomass in water**
		Oxygen demand	**Soil texture (% sand, silt, clay)**	Organic carbon in water layer
		Phosphorus content	Soil texture classification system	Organic content in sediment
		**Total organic carbon (TOC)**	Water holding capacity	Oxygen content
		**Total suspended solids concentration (TSS)**		**Sediment porosity**
		Volatile suspended solids concentration (VSS)		**Sediment texture (% sand, silt, clay)**
			
				
Experimental setup	**pH**	Addition of nutrients	Experimental humidity	Column height
	Reactor configuration	Bioreactor		Initial mass of sediment
	Type of compound addition			
	Solvent for compound addition	Initial amount of sludge in bioreactor		Initial volume of water
	Spike compound structure	Source of liquid matrix		pH in sediment
	Spike concentration	Type of aeration		pH in water
	Surrounding conditions			Redox potential
	**Temperature**			
				
Other	Reference (DOI)			

a Parameters highlighted in bold are recommended to be reported according to OECD guidelines (OECD Test Nos. 303, 307, and 308).^30–32^ The parameter terminologies are as used in enviPath, and detailed descriptions of each parameter are provided in Table S1 of the SI.

### Biotransformation Pathway Visualization

Pathway visualization is important for both human- and machine-readability of biotransformation processes. We describe here generally accepted conventions for pathway visualization that improve human readability and interpretation as well as best practices for facilitating the automated conversion of a pathway depiction into a machine-readable format. We recommend including at least one image visualization of the biotransformation pathway within the article submission in addition to a filled-out BART template.

Image-based representations of pathways should clearly indicate two-dimensional precursor and transformation product structures as nodes in a directed graph. If a pathway is being proposed for precursors that occur as a homologous series (e.g., structurally similar polyfluoroalkyl substances with different fluorinated carbon chain lengths), it is appropriate to abbreviate the repeating unit (e.g., CF_2_) with a chemical formula, where the location of the transformation is still fully visible. However, text-only chemical formula representations are not easily machine-readable (see Section S1.1 in the Supporting Information (SI)), and structures should be fully drawn as standard two-dimensional chemical graphs whenever possible. Compounds (nodes) should be connected using arrows (edges), indicating the direction of reaction. Each edge should represent one enzymatic biotransformation as best as possible and can be annotated with a double arrow if multiple enzymatic steps are suspected. Where transformation product structures cannot be fully resolved using mass spectrometry, the structural change should be reported for the part of the molecule where the transformation, according to MS2 data, most likely has occurred, by highlighting the relevant part of the molecule in square brackets and annotating those with the respective change (e.g., +O). An example of alternative structure representation in biotransformation pathways and BART is provided in Figure S2 in the SI. Additionally, links to external references such as PubChem or Chemical Entities of Biological Interest (ChEBI) should be clearly associated with compounds within the pathway, whenever possible. Finally, compounds that are suspected to be intermediates but are not detected in the experiment should be shown in parentheses or presented in greyscale to designate the compound as a proposed intermediate.

Once published as static images, decoding biotransformation pathways back into computational representations is exceedingly difficult. While there is hope that biotransformation pathways reported in future studies are deposited in a semantically well-annotated form, there is still a need to curate the backlog of literature for reuse and rediscovery. This challenge has driven an increased interest in developing tools for the computational task of converting an image-based description of a molecule into a machine-recognizable format. This task is called optical chemical structure recognition (OCSR).^23^ In recent years, transformer-based AI and machine-learning breakthroughs have ignited interest in image processing and the development of generative models for predicting chemical structures from images.^23,24^ We therefore suggest that biotransformation pathway images reported in future studies should be deposited in a format that is optimal for OCSR extraction. The output quality of OCSR tools is heavily dependent on both the image quality and content modalities, yet no universally accepted standards govern how reaction schemes should be depicted for optimal extraction. To bridge this gap, we propose high-level guidelines (see SI Section S1.1) that enable researchers to both rapidly assess published biotransformation diagrams for OCSR compatibility and guide authors in crafting inherently OCSR-friendly pathway visualizations.

## Relevant Metadata

Metadata, by definition, is “data about data.” Here, we discuss two types of metadata that are relevant for biotransformation pathways and dissipation kinetics. First, we discuss experimental metadata, which we define as data about the experimental test system in which reported pathways and kinetics were observed and the environmental system on which the test system is based. Second, we discuss reaction metadata, which we define as data about the observed biotransformations.

### Experimental Metadata

Biotransformation studies typically use microbial communities sampled from specific types of environments (e.g., soil, surface water, sediments, and activated sludge). Differences in the composition, function, and physiological status of the microbial communities derived from these environmental samples impact the observed outcomes of biotransformation studies, including both observed biotransformation products and biotransformation rate constants.^25–27^ Additional variability in test outcomes results from varying experimental conditions in the laboratory, including temperature, pH, biomass concentration, solids-to-water ratio, or duration of the experiment.^28,29^ To assess trends in how these variable sources of biomass and experimental conditions affect measurable outcomes across studies, it is essential to systematically collect these experimental metadata for each biotransformation experiment.

Although the Organization for Economic Cooperation and Development (OECD) provides detailed requirements for reporting on assessments of the degradability of organic chemicals in water, aquatic sediment, and soil, as well as in sewage treatment plants, many researchers do not report these key parameters in their scientific publications. This lack of consistency can result in sparse data sets with high uncertainty in reported metadata. To work toward more consensus on the types of parameters reported as metadata for biotransformation experiments in scientific literature, our standardized, machine-readable reporting template (BART) contains lists of preselected parameters for specific environments that are aligned with the respective OECD testing guidelines and expert knowledge of biotransformation data reporting. Key parameters that should be considered in the reporting of environmental conditions for chemical biodegradation testing are provided below in Table 1. For a detailed description of the parameters, including recommended units, we refer the reader to Table S1 in SI or the BART documentation.

The parameters listed in Table 1 have been selected because they are often reported in biotransformation studies and suspected or known to affect biotransformation processes across diverse types of organic contaminants.^4,33,34^ We acknowledge, however, that the factors influencing biotransformation are only partially understood and are likely subject to complex interactions. We anticipate that increased data availability will lead to a better understanding of influential factors, requiring the proposed list to be updated. For example, metagenomic sequencing data for experimental systems could be included as additional metadata in the future. As we believe that this process should be community-driven, we invite researchers to suggest and discuss modifications to this list on the BART discussion forum on GitHub (https://github.com/FennerLabs/BART/discussions). We further acknowledge that the proposed parameters do not specifically list water biodegradation studies (OECD 309) as a category. This and other categories, according to user needs, may be introduced in the future.

### Reaction Metadata

Ideally, when reporting biotransformation pathways, each reaction connecting a reactant-product pair should be commented on in terms of the plausibility of the proposed structural changes relative to known enzymatic reaction mechanisms. If available, evidence from enzyme-based studies to support the proposed reactions should be provided. We encourage authors to additionally categorize observed reactions into common types of enzymatically catalyzed transformations (e.g., hydrolysis, reductive dehalogenation). If additional experiments provide evidence about the enzyme responsible for the observed biotransformation, database identifiers for the enzyme should be included (e.g., Rhea, KEGG, EC number, UniProt).

## Biotransformation Kinetics

When single first order kinetics can be assumed, biotransformation kinetics are reported as primary biotransformation rates (k) or half-lives (DT_50_), which describe the disappearance of a substance from the test system over time and are interconvertible:

$$DT_{50}=\frac{\ln\left(2\right)}{k}$$

For regulatory soil and water-sediment biotransformation studies, dissipation kinetics are generally reported as DT_50_, while k is the preferred choice for biotransformation experiments in activated sludge.^26,35^ In some sludge studies, dissipation kinetics are reported as the biomass-corrected rate constant obtained from dividing the observed k by the concentration of total suspended solids (TSS).^36,37^ For experiments in water-sediment systems, separate dissipation half-lives from the water and the sediment phase may be reported, in addition to the dissipation half-life for the total system.^38^ Half-lives and rate constants are calculated from concentration–time series of the parent compound.

For reporting kinetic information, model assumptions and parameters used to calculate dissipation kinetics should be reported, including the quality of the model fit and correction factors (e.g., biomass, adsorption, abiotic processes).^20,33^ If appropriate, an estimation of error such as the standard deviation should be provided as ± error (one standard deviation) next to the reported values. Particularly for water-sediment studies, it is essential to report whether the reported half-lives refer to dissipation from the water or the sediment compartment, or to actual degradation in either of those two compartments or in the total system.^38^ Kinetic information can be reported in BART using the *Kinetics_Confidence* tab, where both half-lives and rate constants can be entered in rows next to their associated compounds as well as information on the model used to calculate each value, any corrections for adsorption/abiotic processes, and the model fit using the R^2^ value. Additionally, we recommend that concentration–time series underlying the reported kinetic information should be provided in a tabular format (csv or Excel file) in the Supporting Information of the manuscripts to ensure reproducibility.

## Benefits of FAIR Data Reporting: The Example of PFAA Precursor Biotransformation

In this section, we show how biotransformation research can benefit from standardized biotransformation data reporting using polyfluoroalkyl substances as an example, as understanding their environmental fate is globally urgent.^39^ Though PFASs, by definition, include both polyfluoroalkyl and perfluoroalkyl substances,^40^ there is a growing need to understand the details of the conversion between these two subclasses through better pathway and kinetics prediction models. This information can be further used to support contaminated site assessments by predicting from which chemical structures and under which circumstances the formation of the highly persistent PFAAs is expected.

The number of studies investigating the biotransformation potential of PFASs has been increasing in recent years due to widespread occurrence of PFAS in the environment and the need to characterize the spatial and temporal distribution of PFASs, including PFAA precursors, at contaminated sites.^8,41–43^ With growing scientific literature on this topic, there is a need to systematically and efficiently synthesize biotransformation data as they become available to ensure our understanding of the fate of PFASs in the environment remains up-to-date as new pathways are discovered. Multiple review papers have conventionally synthesized biotransformation data to discover trends in PFAS biotransformations, yet the information used for such analyses is not easily updated or shared for future machine-learning analyses and model training.^7,8,44–46^ For example, Choi et al.^7^ found that N-dealkylations are key biotransformations for precursors derived from both fluorotelomer and electrochemical fluorination processes by manually summarizing and visualizing observed biotransformation pathways for polyfluoroalkyl substances derived from aqueous film-forming foam in different environmental systems. This is an essential observation for understanding the fate of PFAA precursors in the environment, but the data used to generate this conclusion are not provided in a format that can be easily extracted or modified as new data are generated. Instead, information regarding chemical structures, reaction connectivity, and environmental test conditions contained within the provided pathways must be extracted manually (or by using an OCSR tool in the future), which can be a bottleneck for using this data set for training pathway prediction models. To tackle this issue in existing research, we have extracted data on PFAS biotransformations from the scientific literature and restructured the data into the BART format. We uploaded these data to the enviPath platform to create an online, publicly available, and FAIR database on PFAS biotransformations, called enviPath-PFAS. In the following, we highlight the advantages of using our recommended standard reporting method by showcasing the utility of the newly developed enviPath-PFAS. We note that expert judgment was required for interpreting reported metadata in scientific literature, which could be a source of epistemic uncertainty in our data synthesis.

### Data set Overview

Biotransformation data for the enviPath-PFAS package was collected from 39 scientific publications following a search for literature containing images of PFAS biotransformation pathways (see SI Section S3 and Table S2). As of publication, our data set contains 78 pathways with 351 compounds and 595 reactions. This collection of literature is not yet comprehensive, and we are continually working to expand it with more data. All BART files used to generate the enviPath-PFAS package are openly accessible on Zenodo.^47^ Upon uploading the BART files into enviPath, pathways are stored and visualized as graphs consisting of compounds (nodes) that are connected by biotransformation reactions (edges). Further details on the structure of the enviPath environment are provided elsewhere.^9^ Our data set in enviPath is freely available to the public and can be accessed on envipath.org (https://envipath.org/package/d2d2410f-43e9-401d-b190-20862baed780), or via python using the enviPath-python application programming interface (https://github.com/enviPath/enviPath-python). We invite the scientific community to contribute to this data package by using the BART template with the objective of growing the data set into a comprehensive collection of biotransformation data for PFASs. To do so, one must download an empty BART template from our GitHub repository, fill it out with biotransformation information according to the README file in the repository, and submit the filled-out BART template to the enviPath community forum here: https://community.envipath.org/t/bart-data-submission/92. Each submitted BART template will be reviewed by a member of the enviPath team to ensure that the data are properly represented before being added to the enviPath-PFAS package.

### Example Utility of the enviPath-PFAS Data Package: Analyzing Precursors of PFOA and PFHxA

Ignoring source zones of persistent contaminants has been compared to a perpetually leaking tap where, despite cleanup efforts, the contamination remains, making remediation more costly.^43^ This is one reason why legacy PFAAs are still detected in the environment despite restricting their use since 2006.^48^ The experimental identification of PFAA precursors is particularly challenging given the evidence that many precursors share similar intermediates and end products.^46^ However, mapping all available biotransformation pathways together can make it easier to determine original precursor structures, which is an important application for remediation practitioners.^48^ Here, we illustrate how enviPath-PFAS can be used to identify precursors of both perfluorooctanoic acid (PFOA) and perfluorohexanoic acid (PFHxA). Searching enviPath-PFAS for PFOA and PFHxA will, at present, highlight 12 and 23 documented pathways containing PFOA and PFHxA, which are related to 8 and 15 unique precursors, respectively. This information can be conceptualized as a pathway map that contains all recorded precursors and biotransformation pathways leading to PFOA and PFHxA formation, according to the data contained in enviPath-PFAS as of publication (Figure 2).

**Figure 2:**
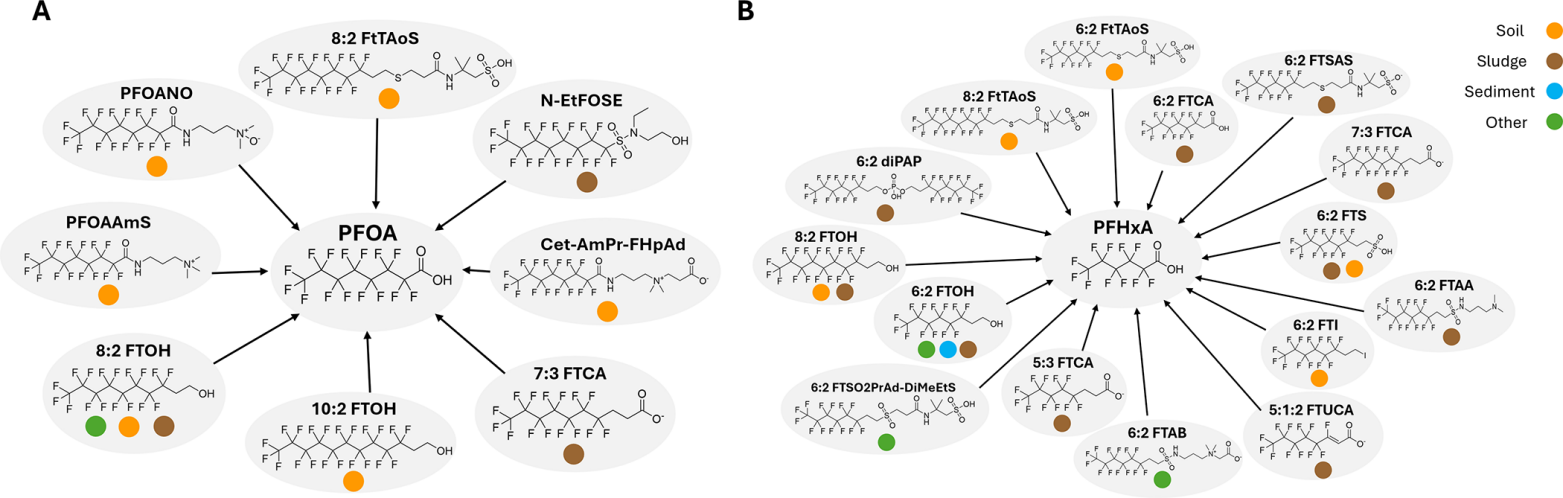
(A) Simplified precursor map manually drawn from eight documented biotransformation pathways of precursors that form PFOA (N-EtFOSE,^49^ Cet-AmPr-FHpAd,^45^ PFOANO,^50^ 8:2 FtTAoS,^51^ 10:2 FTOH,^52^ 8:2 FTOH,^53^ PFOAAmS,^54^ and 7:3 FTCA^20^). (B) Simplified pathway map manually drawn from 15 biotransformation pathways of precursors that form to PFHxA (6:2 diPAP,^55^ 6:2 FTAB,^56^ 6:2 FTCA,^57^ 6:2 FTI,^58^ 6:2 FTOH,^59^ 6:2 FTSA,^60^ 6:2 FTSO2PrAd-DiMeEtS,^61^ 6:2 FtTAoS,^62^ 8:2 FTOH,^53^ 8:2 FtTAoS,^51^ 6:2 FTSAS,^63^ 5:3 FTCA,^20^ 7:3 FTCA,^20^ 6:2 FTAA,^64^ 5:1:2 FTUCA^20^) as observed in experiments with environmentally derived microbial communities. Each colored circle on the precursor nodes represents the type of environmental microbial community in which the precursor was tested: soil (orange), sludge (brown), sediment (blue), and other (green). Intermediates are not shown in the simplified pathway maps; therefore, parent nodes are directly connected to the final end product. Pathways were extracted from studies published from 2000 to 2024.

The manually drawn precursor pathway maps shown in Figure 2A and B highlight the multiple ways that PFOA and PFHxA can be formed in the environment. For instance, in Figure 2A, although there are eight nodes presented as precursors, this representation does not include all intermediates or other compounds that may biotransform into any of the shown precursors. The enviPath-PFAS database provides users a method to easily explore these additional intermediates and precursors due to its built-in data structure in which the biotransformation data are stored as interconnected nodes and edges in a graphical reaction network. This will allow for more comprehensive assessments of PFAA precursors in contaminated site assessments and exemplifies how this approach could be applied to other organic contaminants with persistent transformation products in the broader enviPath database. Additionally, PFOA and PFHxA are part of a homologous series of PFASs, and therefore we expect similar biotransformation reactions in their formation pathways.^65^ As a consequence, the eight-perfluorocarbon versions of the identified precursors to PFHxA may also be inferred to be precursors to PFOA. However, it is important to note that such inferences have limitations, as biotransformations can vary with chain length and not all processes may occur in the same way for PFASs with different chain lengths. This exemplifies the need to consider entire biotransformation pathways for understanding PFAA formation at contaminated sites as there is a potential that many important precursors are overlooked.

## Outlook

A standardized approach to reporting chemical contaminant biotransformation data will provide researchers and practitioners with access to high-quality data sets and pave the way toward better predictions of the fate of chemical contaminants in the environment. We recognize that the proposed scheme for reporting biotransformation data (BART) is only the first version, requiring future adjustments with evolving experimental techniques and new data types that might become relevant in the future; e.g., the rate of *formation* of terminal PFAAs from precursors will be of particular interest and may warrant separate reporting.

We envision that this further development of biotransformation reporting standards should be community-driven, which is why we have published our recommended reporting template on GitHub, where members of the scientific community are invited to provide feedback and suggest adjustments to the template. With this work, we aim to raise awareness of the importance of sharing biotransformation data in a standardized and machine-readable format for building high-quality biotransformation databases and related downstream applications, such as biotransformation prediction. We believe that an effective strategy for data sharing will help to prevent future release of chemical contaminants with the potential to biotransform into products with structures containing properties that are more persistent than those of the parent compound (as in the case for many polyfluoroalkyl substances within the family of PFASs).

## Key Messages

- Biotransformation data reporting needs to be standardized to improve the quality and availability of large, high-quality data sets that can be used for training pathway prediction models.
- We provide a template with a standardized, machine-readable format for reporting biotransformation data (https://github.com/FennerLabs/BART).
- We have created an online, publicly available, and FAIR database on PFAS biotransformations, called enviPath-PFAS (https://envipath.org/package/d2d2410f-43e9-401d-b190-20862baed780)

## Supplementary Material



## Notes

Additional discussion of optical chemical recognition (OCSR) tools with examples of optimal image representations of pathways, examples of alternative structure representation, descriptions of key metadata parameters in the Biotransformation Reporting Tool (BART), and summary of pathways extracted from literature used to create the enviPath-PFAS package (PDF)
