# Supplementary material for: FAIR and Effective Communication of Data on Chemical Contaminant Biotransformation in the Environment
Source: Environ Sci Technol Lett. 2025 Oct 22;12(11):1462–70. doi: 10.1021/acs.estlett.5c00753 (PMC12613814; doi:10.1021/acs.estlett.5c00753)
Supplement: Supplementary file 1 [file ez5c00753_si_001.pdf]

Supporting Information for:

FAIR and Effective Communication of Data on Chemical Contaminant Biotransformation in the Environment

*Stephanie L. Rich[a,b], Jasmin Hafner[a,b], Moritz Salz[a,b], Mojtaba Qanbarzadeh[c], Fanshu Geng[d], Liqing Yan[d], Jinxia Liu[e,f], Damian E. Helbling[d], Christopher P. Higgins[c], and Kathrin Fenner[a,b]**

[a]Department of Chemistry, University of Zürich, 8057, Zürich, Switzerland

[b]Eawag, Swiss Federal Institute of Aquatic Science and Technology, 8600, Dübendorf, Switzerland

[c]Department of Civil and Environmental Engineering, Colorado School of Mines, Golden, CO, 80401, USA

[d]School of Civil and Environmental Engineering, Cornell University, Ithaca, NY, 14850, USA

[e]Department of Civil Engineering, McGill University, Montreal, QC H3A 0C3, Canada

[f]Department of Civil and Environmental Engineering, Hong Kong Polytechnic University, Kowloon, Hong Kong SAR

*Corresponding Author: kathrin.fenner@chem.uzh.ch

Institutional Profile: https://www.eawag.ch/en/about-us/portrait/organisation/staff/profile/kathrin-fenner/show/

Google Scholar Profile: https://scholar.google.com/citations?user=tTti3QsAAAAJ&hl=en

Section S1.1: Biotransformation Pathway Visualization with Optical Chemical Recognition Tools

When biotransformation pathways are drawn in common structure editors, they can be readily exported into machine-readable formats such as MOL-file, SMILES (simplified molecular input line entry specification) or InChI (international chemical identifier). However, once published as static images, decoding them back into computational representations becomes exceedingly difficult. While there is hope that biotransformation pathways reported in future studies are deposited in a semantically well-annotated form, there is still a need to curate the backlog of literature for re-use and re-discovery. The computational task to convert an image-based description of a molecule into a machine-recognizable format is called optical chemical structure recognition (OCSR). The field has advanced dramatically over the past few decades, and especially in recent years, transformer-based AI and machine-learning breakthroughs have ignited interest in image processing and the development of generative models for predicting chemical structures from images.[1] Whereas most OCSR tools remain optimized for single-molecule extraction, only three (OSRA,[2] RxnScribe,[3] and ReactionDataExtractor 2.0 (RDE)[4]) currently support full reaction parsing from images. OSRA relies on a rule-based pipeline that first vectorizes input images and then interprets vectors and nodes as bonds and atoms using predefined heuristic rules. In contrast, RxnScribe and RDE leverage deep-learning (DL) architectures trained on large, manually curated reaction-scheme datasets. In addition to SMILES of reactants and products, RxnScribe and RDE also record connectivity information and reaction conditions. Krasnov et al.[5] demonstrate that RxnScribe delivers the highest reaction-extraction performance (precision: 77%, recall: 99%, F1: 86%) on a hand-curated subset of 103 reaction figures from patent and patent-application images, substantially outperforming OSRA (precision: 64%, recall:

63%, F1: 64%) and RDE (precision: 49%, recall: 60%, F1: 55%). It should be emphasized that these performance results are specific to the testing images, for other diagram types (*e.g.*, PFAS biotransformation pathways), and tool behavior may differ.

The output quality of these tools is also heavily dependent both on the image quality and content modalities, yet no universally accepted standards govern how reaction schemes should be depicted for optimal extraction. To bridge this gap, we propose some high-level guidelines that enable researchers to both rapidly assess published biotransformation diagrams for OCSR compatibility and guide authors in crafting inherently OCSR-friendly pathway illustrations. Because biotransformation schemes are often highly intricate and diverse, these recommendations remain intentionally broad, focusing on key factors to improve automatic extraction across diverse representations.

First, high-resolution, digitally generated figures are essential for optimal OCSR performance. Most OCSR tools rely on a series of low-level image pre-processing steps (*e.g.*, binarization, thinning/skeletonization, noise-reduction, vectorization) that all implicitly assume bonds, letters, and nodes occupy a certain minimum number of pixels.[1] At low resolution, each of these steps could break the process. For example, noise reduction filters that are tuned for 300 dpi can erode genuine features when they are only a few pixels wide. Likewise, DL-based tools suffer a distribution shift when trained on high-dpi diagrams but tested on lower-dpi inputs; DL models are only as good as the data they see during training. Despite efforts like DECIMER to include scanned or hand-drawn figures in the training dataset, output quality often remains unsatisfactory.[6] On top of resolution, many tools (*e.g.*, OSRA and MolScribe, on which RxnScribe relies) use fixed pixel-size thresholds relative to the overall canvas, so a molecule that occupies only a small fraction of a high-dpi image can still be treated as noise and discarded. Therefore, for complex

pathway figures, where one precursor may branch into different pathways and involve tens of steps, researchers might consider drawing each route on its own tightly cropped canvas, ensuring every molecule structure fills enough of the frame to preserve connectivity and legibility.

Beyond image quality, the layout of the reaction scheme profoundly influences OCSR accuracy. Biotransformation pathways can appear as single lines,[7] multi-line sequences,[8] branched trees,[9] and cyclic loops.[10] Figures with a clear left-to-right (or top-to-bottom) progression of reactants, arrows, and products are much easier to parse than densely branched or cyclic graphs. Complex, overlapping branches force the tool to infer an ordering that isn't explicit, driving up ambiguity and error rates. Arrows serve as implicit delimiters between reactants, conditions, and products. Straight, single-headed arrows that clearly separate reactants from products are ideal. Rule-based methods often fail on vertical, branched, or curved arrows, and even RxnScribe — despite being trained on some branched and cyclic examples—can struggle when arrows loop, branch, or curve in unexpected ways.[3] Equally important is how molecules themselves are rendered. Standard 2D chemical graphs—lines and wedges denoting bonds—yield the most reliable results, because entity detectors cluster bond-rich regions of predictable size and shape. By contrast, figures that use text-only labels, mixing text/structure format, ball-and-stick (or other 3D rendering), or R-groups or homologous series where R or n are defined elsewhere can lead to missed or mis-assigned entities. Moreover, colors are widely used in biotransformation pathways to highlight active sites or differentiate various reaction routes. This is great for human readability but might cause issues for OCSR extraction because most OCSR tools start by converting the image to greyscale, applying quantitative metrics such as luminance. Anything with a luminance above 200 (such as lavender, cyan, light pink, mint green, etc.) will likely sit very close to the white background (luminance of 255) and risk being discarded or fragmented by thresholding and

noise filters. Researchers should aim for colors with much lower luminance (e.g., black, dark blues, dark greens, dark grey, etc.) if color is needed.

To support the guidelines discussed above, we compare OCSR tool performance on two representations of the same example PFAS biotransformation pathway, one optimized for OCSR and one deliberately challenging for OCSR. **Figure S1(a)** exemplifies the optimized version, with straight arrows, black-and-white skeletal structures, and a clear, mostly linear layout. In contrast, **Figure S1(b)** shows the challenging version, featuring high-luminance colors, curved nonstandard arrows, and mixed structural/formula depictions. None of the tested tools could extract correct SMILES from the challenging version (**Fig. S1(c)**). In comparison, RxnScribe successfully identified all molecular structures and 10 out of 13 reaction steps in the optimized version. OSRA and RDE are also capable of identifying the majority of the molecular structures in the optimized version. These results highlight that maintaining simple, conventional reaction arrows and molecular drawings greatly improves the ability of OCSR pipelines—whether rule-based or machine-learning-driven—to accurately reconstruct chemical information.

(a)

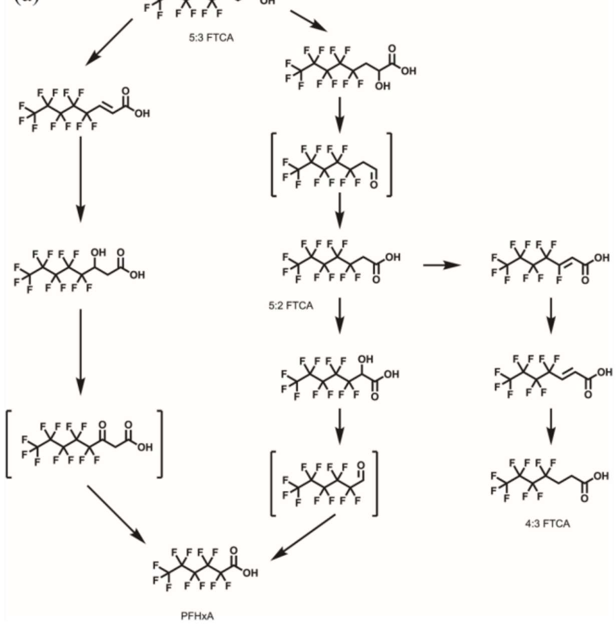

(b)

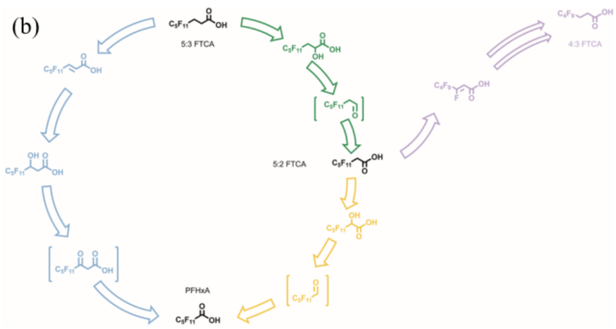

(c)

Extraction performance of three OCSR tools on optimized and challenging examples

	# Correct molecules		# Correctly steps	
	Good	Bad	Good	Bad
Truth	13	13	13	13
OSRA	11	0	NA	NA
RxnScrib	13	0	10	0
RDE	11	0	6	0

Fig. S1 (a) a biotransformation pathway for 5:3 FTCA optimized for OCSR tool performance**; (b)** a biotransformation pathway for 5:3 FTCA designed to challenge OCSR tool performance**; (c)** extraction performance of three OCSR tools on optimized (Good) and challenging (Bad) versions.

Section 1.2: Alternative Structure Representation

[A]

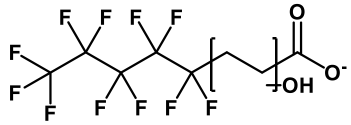

5:3 FTCA_357
(Pathway Representation)

[B]

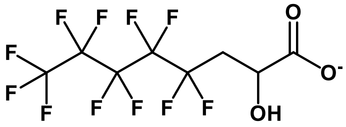

5:3 FTCA_357
(Reported Structure)

[C]

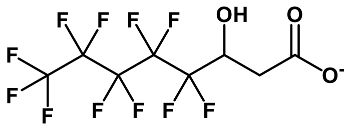

5:3 FTCA_357
(Alternative Structure 1)

Figure S2: Example of alternative structure representation, where structure **[A]** shows how a compound with alternatives structures can be reported in a pathway image, structure **[B]** shows a main structure to report using SMILES, and structure **[C]** is the first alternative structure indicating the other possible location of where the observed hydroxylation might have occurred.

Section S2: Description of Key Metadata Parameters

Table S1: List of metadata parameters including a detailed explanation of each parameter as well as an example of the parameter in question and typical units used to report the parameters.

Parameter	Definition	Example or Units
Addition of nutrients	Ingredients added to media to assist microbial growth	Potassium phosphate
Ammonia uptake rate	Rate at which microorganisms convert ammonia	mg NH4/L-h
Biological treatment technology	The process for which the full-scale wastewater treatment system is designed	nitrification
Bioreactor	Type of reactor used as an experimental test system	120 mL glass batch reactors
Bulk density	Density of the bulk test medium (soil)	g/cm3
Cation exchange capacity	Measure of a soil's ability to hold and exchange positively charged ions	meq/100g
Column height	Test column height in water-sediment experiments	cm
Dissolved organic carbon	Concentration of dissolved organic carbon in the test system	mg C/L
Dissolved oxygen concentration	Concentration of dissolved oxygen in the test system	mg/L
Experimental humidity	Humidity in test soil in percent of water storage capacity of test soil (see below)	%
Initial amount of sludge in bioreactor	Volume of activated sludge in batch reactors at the start of the experiment	mL
Initial mass of sediment	Either wet or dry mass of sediment at the start of the experiment	g
Initial volume of water	Volume of water in batch reactors at the start of the experiment	mL
Inoculum source	Description of where biological inoculum was sampled	activated sludge from aerated nitrifying treatment basin
Microbial biomass	Amount of biomass per gram of soil in soil experiments	µg C/g soil
Microbial biomass in sediment	Amount of biomass per gram of sediment in water-sediment experiments	cells/g sediment
Microbial biomass in water	Number of cells present in water for water-sediment experiments	cells/mL
Nitrogen content	Concentration of NH4-N or Total Nitrogen in batch reactors	mg/L
Non-extractable residue (NER)	Fraction of the parent compound that is immobilized to solid matrices	%
Organic carbon in water layer	Either total or dissolved organic carbon in water layer for water-sediment experiments	mg/L
Organic content	Either organic matter or organic carbon in batch reactors	%
Organic content in sediment	Either organic matter or organic carbon in sediment layer for water-sediment experiments	%
Oxygen content	Concentration of oxygen in experimental reactors	mg/L
Oxygen demand	Either chemical or biological oxygen demand in experimental reactors	mg/L
pH	Hydrogen ion activity in test system	-
pH in sediment	Hydrogen ion activity in sediment	-
pH in water	Hydrogen ion activity in water	-
Phosphorous content	Concentration of phosphorus in test system	mg/L

Purpose of WWTP	Description of the type of wastewater treatment plant in the study	municipal WW
Reactor configuration	Indicates if experimental reactor is open or closed to the atmosphere	Open or closed
Redox condition	Description of the redox condition in experimental reactors	aerobic
Redox potential	Redox potential in water-sediment systems	mV
Reference (DOI)	Digital object identifier for the paper with the pathway	10.1186/s13321-024-00881-6
Sample location	Description of the location where the sample was taken	Zürich, Switzerland
Sampling depth	Depth of sample taken for soil experiments	cm
Sediment origin	Description of where sediment sample was taken	Greifensee, Switzerland
Sediment porosity	Porosity of the sediment	-
Sediment texture (% sand, silt, clay)	Description of sediment texture with % sand, silt, and/or clay	%
Sludge retention time	Either sludge age or solids retention time at the sampled wastewater treatment plant	d
Soil origin	Description of where soil sample was taken	Zürich, Switzerland
Soil texture (% sand, silt, clay)	Description of soil texture with % sand, silt, and/or clay	%
Soil texture classification system	Name of classification system used to describe soil texture	USDA
Solvent for compound addition	Solvent used in stock solution for spike compound	MeOH
Source of liquid matrix	Description of where liquid matrix was sampled from	Aeration basin
Spike compound structure	SMILES of the compound spiked into the batch reactors	CN1C=NC2=C1C(=O)N(C(=O)N2C)C
Spike concentration	Nominal or measured concentration of the tested chemical at the start of the spike experiment	µg/L
Surrounding conditions	Indicates if experimental system is in a light or dark test environment	Light or dark
Temperature	Temperature of test experiment	°C
Total organic carbon (TOC)	Total concentration of organic carbon in the test system	mg C/L
Total suspended solids concentration (TSS)	Concentration of suspended solids in the test system	g/L
Type of aeration	Manner in which batch reactors are aerated	shaking
Type of compound addition	Manner in which the spike compound was added	spiking in solvent
Volatile suspended solids concentration (VSS)	Concentration of volatile suspended solids in the test system	g/L
Water holding capacity	Water holding capacity in soil test systems	%

Section S3: Summary of pathways used to generate data in enviPath-PFAS package.

Table S2: List of reference authors & year, DOI, spiked PFAS, and environment used for inoculum for all 78 pathways from the 39 papers used to generate the enviPath-PFAS package. [9–46]

Authors & Year	DOI	Spiked PFAS	Environment
Arakaki, A. et al 2010	10.1007/s00253-010-2815-9	DTFA	Sludge
Benskin, J.P. et al 2013	10.1021/es304336r	SAmPAP Diester	Sediment
Butt, C. et al 2010	10.1021/es100702a	8:2 FTOH	Rainbow Trout
Che, S. et al 2021	10.1021/acs.est.1c05509	3,3,3 TFPrA	Sludge
Che, S. et al 2021	10.1021/acs.est.1c05509	4,5,5-TF-4-enoic Acid	Sludge
Che, S. et al 2021	10.1021/acs.est.1c05509	5,5,5-TFPeA	Sludge
Chen, H. et al 2020	10.1021/acs.estlett.0c00543	PFOANO	Soil
Cook, E. et al 2022	10.1021/acs.est.2c00261	AmPr-FHxSA	Soil
Dagostino, L.A. & Maybury, S.A. et al 2017	10.1002/etc.3750	6:2 FTAA	Sludge
Dasu, K. & Lee, L.S. 2012	10.1021/es203978g	8:2 FTS	Soil
Dasu, K. & Lee, L.S. 2016	10.1016/j.chemosphere.2015.11.021	8:2 FTU	Soil
Dasu, K. & Lee, L.S. 2016	10.1016/j.chemosphere.2015.11.021	8:2 HMU	Soil
Dasu, K. & Lee, L.S. 2016	10.1016/j.chemosphere.2015.11.021	8:2 FTOH	Soil
Dong, S. & Yan, P. et al 2024	10.1021/acs.est.4c08665	TAmPr-FBSA-AA	Soil
Dong, S. & Yan, P. et al 2024	10.1021/acs.est.4c08665	TAmPr-FPeSA-AA	Soil
Dong, S. & Yan, P. et al 2024	10.1021/acs.est.4c08665	TAmPr-FHxSA-AA	Soil
Dong, S. & Yan, P. et al 2024	10.1021/acs.est.4c08665	AmPr-FBSA-PrA	Soil
Dong, S. & Yan, P. et al 2024	10.1021/acs.est.4c08665	AmPr-FPeSA-PrA	Soil
Dong, S. & Yan, P. et al 2024	10.1021/acs.est.4c08665	AmPr-FHxSA-PrA	Soil
Dong, S. & Yan, P. et al 2024	10.1021/acs.est.4c08665	AmPr-FHpSA-PrA	Soil
Dong, S. & Yan, P. et al 2024	10.1021/acs.est.4c08665	AmPr-FOSA-PrA	Soil
Dong, S. & Yan, P. et al 2024	10.1021/acs.est.4c08665	CEt-AmPr-FBSA-PrA	Soil
Dong, S. & Yan, P. et al 2024	10.1021/acs.est.4c08665	Cet-AmPr-FHpAd	Soil
Fang, B. et al 2024	10.1021/acs.est.3c05506	6:2 FTNO	Sludge
Fang, B. et al 2024	10.1021/acs.est.3c05506	6:2 FTSA	Sludge
Geng & Helbling 2024	10.1021/acs.est.4c09534	1:3 FTCA	Sludge
Geng & Helbling 2024	10.1021/acs.est.4c09534	1:4 FTCA	Sludge
Geng & Helbling 2024	10.1021/acs.est.4c09534	3:3 FTCA	Sludge
Geng & Helbling 2024	10.1021/acs.est.4c09534	5:3 FTCA	Sludge
Geng & Helbling 2024	10.1021/acs.est.4c09534	7:3 FTCA	Sludge
Geng & Helbling 2024	10.1021/acs.est.4c09534	5:1:2 FTUCA	Sludge
Geng & Helbling 2024	10.1021/acs.est.4c09534	4:2 FTS	Sludge
Geng & Helbling 2024	10.1021/acs.est.4c09534	6:2 FTS	Sludge
Harding-Marjanovic, K.C. et al 2015	10.1021/acs.est.5b01219	4:2 FtTAoS	Soil
Harding-Marjanovic, K.C. et al 2015	10.1021/acs.est.5b01219	6:2 FTTAoS	Soil
Harding-Marjanovic, K.C. et al 2015	10.1021/acs.est.5b01219	8:2 FTTAoS	Soil
Jin, B. et al 2023	10.1021/acs.estlett.3c00411	2-(trifluoromethoxy)acetic acid (E1)	Sludge
Jin, B. et al 2023	10.1021/acs.estlett.3c00411	3-(trifluoromethoxy)propanoic acid (E5)	Sludge
Jin, B. et al 2023	10.1021/acs.estlett.3c00411	2-(perfluoroethoxy)acetic acid (E7)	Sludge
Joudan, S. et al 2022	10.1039/d1em00358e	diFESOS	Sludge
LaFond, J. et al 2024	10.1021/acs.est.4c01931	SPrAmPr-FHxSAPrS	Groundwater
LaFond, J. et al 2024	10.1021/acs.est.4c01931	S-OHPrAmPr-FHxSA-OHPrS	Groundwater
LaFond, J. et al 2024	10.1021/acs.est.4c01931	SPrAmPr-FHxSAA	Groundwater
LaFond, J. et al 2024	10.1021/acs.est.4c01931	SPrAmPr-FHxSA	Groundwater

LaFond, J. et al 2024	10.1021/acs.est.4c01931	S-OHPrAmPr-FHxSA	Groundwater
LaFond, J. et al 2024	10.1021/acs.est.4c01931	AmPr-FHxSA	Groundwater
LaFond, J. et al 2024	10.1021/acs.est.4c01931	SPr-FHxSA	Groundwater
LaFond, J. et al 2024	10.1021/acs.est.4c01931	6:2 FTSO2PrAd-DiMeEtS	Groundwater
LaFond, J. et al 2024	10.1021/acs.est.4c01931	EtFOSE	Groundwater
Lange, C. et al 2000	https://static.ewg.org/reports/2003/pfcs/226-1030a078.pdf	N-EtFOSE	Sludge
Lee, H. et al 2010	10.1021/es9028183	4:2 monoPAP	Sludge
Lee, H. et al 2010	10.1021/es9028183	6:2 diPAP	Sludge
Lee, H. et al 2010	10.1021/es9028183	8:2 monoPAP	Sludge
Lee, H. et al 2010	10.1021/es9028183	10:2 monoPAP	Sludge
Li, F. et al 2018	10.1016/j.chemosphere.2018.02.065	8:2 FTOH	Sludge
Liu, J. et al 2010 a	10.1016/j.chemosphere.2009.10.044	6:2 FTOH	Soil
Liu, J. et al 2019	10.1016/j.scitotenv.2018.09.214	N-EtFOSE	Soil
Liu, M. et al 2021	10.1021/acs.est.0c05811	PFOSB	Soil
Mejia-Avendaño, S. et al 2015	10.1016/j.chemosphere.2014.09.059	N-EtFOSE	Soil
Mejia-Avendaño, S. et al 2016	10.1021/acs.est.6b00140	PFOAAmS	Soil
Mejia-Avendaño, S. et al 2016	10.1021/acs.est.6b00140	PFOSAmS	Soil
Olivares, C. et al 2022	10.1039/d1em00494h	6:2 FtTAoS	Soil
Rhoads, K.R. et al 2008	10.1021/es702866c	N-EtFOSE	Sludge
Royer, L.A. et al 2015	10.1016/j.chemosphere.2014.09.077	8:2 FTAC	Soil
Royer, L.A. et al 2015	10.1016/j.chemosphere.2014.09.077	8:2 FTMAC	Soil
Ruan, T. et al 2013	10.1021/es4018128	6:2 FTI	Soil
Ruyle, B. et al 2023	10.1021/acs.est.2c07178	PFHxSAmS	Sediment
Shaw, D.M.J. et al 2019	10.1016/j.scitotenv.2018.08.012	6:2 FTAB	Pure Culture
Wang, N. et al 2009	10.1016/j.chemosphere.2009.01.033	8:2 FTOH	Soil
Wang, N. et al 2011	10.1016/j.chemosphere.2010.11.003	6:2 FTSA	Sludge
Wang, N. et al 2012	10.1016/j.chemosphere.2011.12.056	5:3 FTCA	Sludge
Weiner, B. et al 2013	10.1071/EN13128	6:2 FTSAS	Sludge
Wu, C. et al 2024	10.1016/j.watres.2024.121431	6:2 FTCA	Sludge
Yan, P. et al 2024	10.1016/j.watres.2023.120941	6:2 FTS	Soil
Zhang, S. et al 2013	10.1021/es4000824	6:2 FTOH	Sludge
Zhang, S. et al 2013	10.1021/es4000824	8:2 FTOH	Sludge
Zhao, L. et al 2013	10.1016/j.chemosphere.2012.06.035	6:2 FTOH	Sediment
Zhao, S. et al 2017	10.1016/j.envpol.2016.09.030	10:2 FTOH	Earthworm/Soil

References

(1) Rajan, K.; Brinkhaus, H. O.; Zielesny, A.; Steinbeck, C. A Review of Optical Chemical Structure Recognition Tools. *Journal of Cheminformatics*. BioMed Central Ltd October 7, 2020. https://doi.org/10.1186/s13321-020-00465-0.

(2) Filippov, I. V.; Nicklaus, M. C. Optical Structure Recognition Software to Recover Chemical Information: OSRA, an Open Source Solution. *J Chem Inf Model* **2009**, *49* (3), 740–743. https://doi.org/10.1021/ci800067r.

(3) Qian, Y.; Guo, J.; Tu, Z.; Coley, C. W.; Barzilay, R. RxnScribe: A Sequence Generation Model for Reaction Diagram Parsing. *J Chem Inf Model* **2023**, *63* (13), 4030–4041. https://doi.org/10.1021/acs.jcim.3c00439.

(4) Wilary, D. M.; Cole, J. M. ReactionDataExtractor 2.0: A Deep Learning Approach for Data Extraction from Chemical Reaction Schemes. *J Chem Inf Model* **2023**, *63* (19), 6053–6067. https://doi.org/10.1021/acs.jcim.3c00422.

(5) Krasnov, A.; Barnabas, S. J.; Boehme, T.; Boyer, S. K.; Weber, L. Comparing Software Tools for Optical Chemical Structure Recognition. *Digital Discovery* **2024**. https://doi.org/10.1039/d3dd00228d.

(6) Rajan, K.; Brinkhaus, H. O.; Zielesny, A.; Steinbeck, C. Advancements in Hand-Drawn Chemical Structure Recognition through an Enhanced DECIMER Architecture. *J Cheminform* **2024**, *16* (1). https://doi.org/10.1186/s13321-024-00872-7.

(7) Yu, Y.; Che, S.; Ren, C.; Jin, B.; Tian, Z.; Liu, J.; Men, Y. Microbial Defluorination of Unsaturated Per- and Polyfluorinated Carboxylic Acids under Anaerobic and Aerobic Conditions: A Structure Specificity Study. *Environ Sci Technol* **2022**, *56* (8), 4894–4904. https://doi.org/10.1021/acs.est.1c05509.

(8) Chen, Y. J.; Wang, R. De; Shih, Y. L.; Chin, H. Y.; Lin, A. Y. C. Emerging Perfluorobutane Sulfonamido Derivatives as a New Trend of Surfactants Used in the Semiconductor Industry. *Environ Sci Technol* **2024**, *58* (3), 1648–1658. https://doi.org/10.1021/acs.est.3c04435.

(9) Fang, B.; Zhang, Y.; Chen, H.; Qiao, B.; Yu, H.; Zhao, M.; Gao, M.; Li, X.; Yao, Y.; Zhu, L.; Sun, H. Stability and Biotransformation of 6:2 Fluorotelomer Sulfonic Acid, Sulfonamide Amine Oxide, and Sulfonamide Alkylbetaine in Aerobic Sludge. *Environ Sci Technol* **2024**, *58* (5), 2446–2457. https://doi.org/10.1021/acs.est.3c05506.

(10) Geng, F.; Helbling, D. E. Cascading Pathways Regulate the Biotransformations of Eight Fluorotelomer Acids Performed by Wastewater Microbial Communities. *Environ Sci Technol* **2024**. https://doi.org/10.1021/acs.est.4c09534.

(11) Arakaki, A.; Ishii, Y.; Tokuhisa, T.; Murata, S.; Sato, K.; Sonoi, T.; Tatsu, H.; Matsunaga, T. Microbial Biodegradation of a Novel Fluorotelomer Alcohol, 1H,1H,2H,2H,8H,8H-Perfluorododecanol, Yields Short Fluorinated Acids. *Appl Microbiol Biotechnol* **2010**, *88* (5), 1193–1203. https://doi.org/10.1007/s00253-010-2815-9.

(12) Benskin, J. P.; Ikonomou, M. G.; Gobas, F. A. P. C.; Begley, T. H.; Woudneh, M. B.; Cosgrove, J. R. Biodegradation of N-Ethyl Perfluorooctane Sulfonamido Ethanol (EtFOSE) and EtFOSE-Based Phosphate Diester (SAmPAP Diester) in Marine Sediments. *Environ Sci Technol* **2013**, *47* (3), 1381–1389. https://doi.org/10.1021/es304336r.

(13) Butt, C. M.; Muir, D. C. G.; Mabury, S. A. Elucidating the Pathways of Poly- and Perfluorinated Acid Formation in Rainbow Trout. *Environ Sci Technol* **2010**, *44* (13), 4973–4980. https://doi.org/10.1021/es100702a.

(14) Chen, H.; Liu, M.; Munoz, G.; Duy, S. V.; Sauvé, S.; Yao, Y.; Sun, H.; Liu, J. Fast Generation of Perfluoroalkyl Acids from Polyfluoroalkyl Amine Oxides in Aerobic Soils. *Environ Sci Technol Lett* **2020**, *7* (10), 714–720. https://doi.org/10.1021/acs.estlett.0c00543.

(15) Cook, E. K.; Olivares, C. I.; Antell, E. H.; Yi, S.; Nickerson, A.; Choi, Y. J.; Higgins, C. P.; Sedlak, D. L.; Alvarez-Cohen, L. Biological and Chemical Transformation of the Six-Carbon Polyfluoroalkyl Substance N-Dimethyl Ammonio Propyl Perfluorohexane Sulfonamide (AmPr-FHxSA). *Environ Sci Technol* **2022**, *56* (22), 15478–15488. https://doi.org/10.1021/acs.est.2c00261.

(16) D'Agostino, L. A.; Mabury, S. A. Aerobic Biodegradation of 2 Fluorotelomer Sulfonamide–Based Aqueous Film–Forming Foam Components Produces Perfluoroalkyl Carboxylates. *Environ Toxicol Chem* **2017**, *36* (8), 2012–2021. https://doi.org/10.1002/etc.3750.

(17) Dasu, K.; Liu, J.; Lee, L. S. Aerobic Soil Biodegradation of 8:2 Fluorotelomer Stearate Monoester. *Environ Sci Technol* **2012**, *46* (7), 3831–3836. https://doi.org/10.1021/es203978g.

(18) Dasu, K.; Lee, L. S. Aerobic Biodegradation of Toluene-2,4-Di(8:2 Fluorotelomer Urethane) and Hexamethylene-1,6-Di(8:2 Fluorotelomer Urethane) Monomers in Soils. *Chemosphere* **2016**, *144*, 2482–2488. https://doi.org/10.1016/j.chemosphere.2015.11.021.

(19) Dong, S.; Yan, P.-F.; Manz, K. E.; Abriola, L. M.; Pennell, K. D.; Cápiro, N. L. Fate and Transformation of 15 Classes of Per- and Polyfluoroalkyl Substances in Aqueous Film-Forming Foam (AFFF)-Amended Soil Microcosms. *Environ Sci Technol* **2024**. https://doi.org/10.1021/acs.est.4c08665.

(20) Harding-Marjanovic, K. C.; Houtz, E. F.; Yi, S.; Field, J. A.; Sedlak, D. L.; Alvarez-Cohen, L. Aerobic Biotransformation of Fluorotelomer Thioether Amido Sulfonate (Lodyne) in AFFF-Amended Microcosms. *Environ Sci Technol* **2015**, *49* (13), 7666–7674. https://doi.org/10.1021/acs.est.5b01219.

(21) Jin, B.; Zhu, Y.; Zhao, W.; Liu, Z.; Che, S.; Chen, K.; Lin, Y. H.; Liu, J.; Men, Y. Aerobic Biotransformation and Defluorination of Fluoroalkylether Substances (Ether PFAS): Substrate Specificity, Pathways, and Applications. *Environ Sci Technol Lett* **2023**, *10* (9), 755–761. https://doi.org/10.1021/acs.estlett.3c00411.

(22) Joudan, S.; Mabury, S. A. Aerobic Biotransformation of a Novel Highly Functionalized Polyfluoroether-Based Surfactant Using Activated Sludge from a Wastewater Treatment Plant. *Environ Sci Process Impacts* **2022**, *24* (1), 62–71. https://doi.org/10.1039/d1em00358e.

(23) LaFond, J. A.; Rezes, R.; Shojaei, M.; Anderson, T.; Jackson, W. A.; Guelfo, J. L.; Hatzinger, P. B. Biotransformation of PFAA Precursors by Oxygenase-Expressing Bacteria in AFFF-Impacted Groundwater and in Pure-Compound Studies with 6:2 FTS and EtFOSE. *Environ Sci Technol* **2024**, *58* (31), 13820–13832. https://doi.org/10.1021/acs.est.4c01931.

(24) Lange, C. C. *The Aerobic Biodegradation of N-EtFOSE Alcohol by the Microbial Activity Present in Municipal Wastewater Treatment Sludge*; St. Paul, MN, 2000.

(25) Lee, H.; Deon, J.; Mabury, S. A. Biodegradation of Polyfluoroalkyl Phosphates as a Source of Perfluorinated Acids to the Environment. *Environ Sci Technol* **2010**, *44* (9), 3305–3310. https://doi.org/10.1021/es9028183.

(26) Li, F.; Su, Q.; Zhou, Z.; Liao, X.; Zou, J.; Yuan, B.; Sun, W. Anaerobic Biodegradation of 8:2 Fluorotelomer Alcohol in Anaerobic Activated Sludge: Metabolic Products and Pathways. *Chemosphere* **2018**, *200*, 124–132. https://doi.org/10.1016/j.chemosphere.2018.02.065.

(27) Liu, J.; Wang, N.; Szostek, B.; Buck, R. C.; Panciroli, P. K.; Folsom, P. W.; Sulecki, L. M.; Bellin, C. A. 6-2 Fluorotelomer Alcohol Aerobic Biodegradation in Soil and Mixed Bacterial Culture. *Chemosphere* **2010**, *78* (4), 437–444. https://doi.org/10.1016/j.chemosphere.2009.10.044.

(28) Liu, M.; Munoz, G.; Vo Duy, S.; Sauvé, S.; Liu, J. Stability of Nitrogen-Containing Polyfluoroalkyl Substances in Aerobic Soils. *Environ Sci Technol* **2021**, *55* (8), 4698–4708. https://doi.org/10.1021/acs.est.0c05811.

(29) Mejia Avendaño, S.; Liu, J. Production of PFOS from Aerobic Soil Biotransformation of Two Perfluoroalkyl Sulfonamide Derivatives. *Chemosphere* **2015**, *119*, 1084–1090. https://doi.org/10.1016/j.chemosphere.2014.09.059.

(30) Rhoads, K.; Janssen, E.; Luthy, R.; Criddle, C. Aerobic Biotransformation and Fate of N-Ethyl Perfluorooctane Sulfonamidoethanol (N-EtFOSE) in Activated Sludge. *Environ Sci Technol* **2008**, *42* (8), 2873–2878. https://doi.org/10.1021/es702866c.

(31) Royer, L. A.; Lee, L. S.; Russell, M. H.; Nies, L. F.; Turco, R. F. Microbial Transformation of 8:2 Fluorotelomer Acrylate and Methacrylate in Aerobic Soils. *Chemosphere* **2015**, *129*, 54–61. https://doi.org/10.1016/j.chemosphere.2014.09.077.

(32) Ruan, T.; Szostek, B.; Folsom, P. W.; Wolstenholme, B. W.; Liu, R.; Liu, J.; Jiang, G.; Wang, N.; Buck, R. C. Aerobic Soil Biotransformation of 6:2 Fluorotelomer Iodide. *Environ Sci Technol* **2013**, *47* (20), 11504–11511. https://doi.org/10.1021/es4018128.

(33) Shaw, D. M. J.; Munoz, G.; Bottos, E. M.; Duy, S. V.; Sauvé, S.; Liu, J.; Van Hamme, J. D. Degradation and Defluorination of 6:2 Fluorotelomer Sulfonamidoalkyl Betaine and 6:2 Fluorotelomer Sulfonate by Gordonia Sp. Strain NB4-1Y under Sulfur-Limiting Conditions. *Science of the Total Environment* **2019**, *647*, 690–698. https://doi.org/10.1016/j.scitotenv.2018.08.012.

(34) Wang, N.; Szostek, B.; Buck, R. C.; Folsom, P. W.; Sulecki, L. M.; Gannon, J. T. 8-2 Fluorotelomer Alcohol Aerobic Soil Biodegradation: Pathways, Metabolites, and Metabolite Yields. *Chemosphere* **2009**, *75* (8), 1089–1096. https://doi.org/10.1016/j.chemosphere.2009.01.033.

(35) Wang, N.; Liu, J.; Buck, R. C.; Korzeniowski, S. H.; Wolstenholme, B. W.; Folsom, P. W.; Sulecki, L. M. 6:2 Fluorotelomer Sulfonate Aerobic Biotransformation in Activated Sludge of Waste Water Treatment Plants. *Chemosphere* **2011**, *82* (6), 853–858. https://doi.org/10.1016/j.chemosphere.2010.11.003.

(36) Wang, N.; Buck, R. C.; Szostek, B.; Sulecki, L. M.; Wolstenholme, B. W. 5:3 Polyfluorinated Acid Aerobic Biotransformation in Activated Sludge via Novel "One-Carbon Removal Pathways." *Chemosphere* **2012**, *87* (5), 527–534. https://doi.org/10.1016/j.chemosphere.2011.12.056.

(37) Weiner, B.; Yeung, L. W. Y.; Marchington, E. B.; D'Agostino, L. A.; Mabury, S. A. Organic Fluorine Content in Aqueous Film Forming Foams (AFFFs) and Biodegradation of the Foam Component 6 : 2 Fluorotelomermercaptoalkylamido Sulfonate (6 : 2 FTSAS). *Environmental Chemistry* **2013**, *10* (6), 486. https://doi.org/10.1071/EN13128.

(38) Wu, C.; Goodrow, S.; Chen, H.; Li, M. Distinctive Biotransformation and Biodefluorination of 6:2 versus 5:3 Fluorotelomer Carboxylic Acids by Municipal Activated Sludge. *Water Res* **2024**, *254*. https://doi.org/10.1016/j.watres.2024.121431.
(39) Yan, P. F.; Dong, S.; Manz, K. E.; Woodcock, M. J.; Liu, C.; Mezzari, M. P.; Abriola, L. M.; Pennell, K. D.; Cápiro, N. L. Aerobic Biotransformation of 6:2 Fluorotelomer Sulfonate in Soils from Two Aqueous Film-Forming Foam (AFFF)-Impacted Sites. *Water Res* **2024**, *249*. https://doi.org/10.1016/j.watres.2023.120941.
(40) Zhang, S.; Szostek, B.; McCausland, P. K.; Wolstenholme, B. W.; Lu, X.; Wang, N.; Buck, R. C. 6:2 and 8:2 Fluorotelomer Alcohol Anaerobic Biotransformation in Digester Sludge from a WWTP under Methanogenic Conditions. *Environ Sci Technol* **2013**, *47* (9), 4227–4235. https://doi.org/10.1021/es4000824.
(41) Zhao, L.; Folsom, P. W.; Wolstenholme, B. W.; Sun, H.; Wang, N.; Buck, R. C. 6:2 Fluorotelomer Alcohol Biotransformation in an Aerobic River Sediment System. *Chemosphere* **2013**, *90* (2), 203–209. https://doi.org/10.1016/j.chemosphere.2012.06.035.
(42) Zhao, S.; Zhu, L. Uptake and Metabolism of 10:2 Fluorotelomer Alcohol in Soil-Earthworm (Eisenia Fetida) and Soil-Wheat (Triticum Aestivum L.) Systems. *Environmental Pollution* **2017**, *220*, 124–131. https://doi.org/10.1016/j.envpol.2016.09.030.
(43) Liu, J.; Zhong, G.; Li, W.; Mejia Avendaño, S. Isomer-Specific Biotransformation of Perfluoroalkyl Sulfonamide Compounds in Aerobic Soil. *Science of the Total Environment* **2019**, *651*, 766–774. https://doi.org/10.1016/j.scitotenv.2018.09.214.
(44) Che, S.; Jin, B.; Liu, Z.; Yu, Y.; Liu, J.; Men, Y. Structure-Specific Aerobic Defluorination of Short-Chain Fluorinated Carboxylic Acids by Activated Sludge Communities. *Environ Sci Technol Lett* **2021**, *8* (8), 668–674. https://doi.org/10.1021/acs.estlett.1c00511.
(45) Ruyle, B. J.; Schultes, L.; Akob, D. M.; Harris, C. R.; Lorah, M. M.; Vojta, S.; Becanova, J.; McCann, S.; Pickard, H. M.; Pearson, A.; Lohmann, R.; Vecitis, C. D.; Sunderland, E. M. Nitrifying Microorganisms Linked to Biotransformation of Perfluoroalkyl Sulfonamido Precursors from Legacy Aqueous Film-Forming Foams. *Environ Sci Technol* **2023**, *57* (14), 5592–5602. https://doi.org/10.1021/acs.est.2c07178.
(46) Mejia-Avendaño, S.; Duy, S. V.; Sauvé, S.; Liu, J. Generation of Perfluoroalkyl Acids from Aerobic Biotransformation of Quaternary Ammonium Polyfluoroalkyl Surfactants. *Environ Sci Technol* **2016**, *50* (18), 9923–9932. https://doi.org/10.1021/acs.est.6b00140.
